# Prevalence and associated factors of stunting and thinness among adolescent students in Finote Selam Town, Northwest Ethiopia

**DOI:** 10.1186/s41043-021-00269-4

**Published:** 2021-10-18

**Authors:** Damitie Kebede, Reddy P. C. J. Prasad, Degnet Teferi Asres, Hayat Aragaw, Eyerusalem Worku

**Affiliations:** 1 Department of Applied Human Nutrition, Faculty of Chemical and Food Engineering, Bahir Dar Institute of Technology, Bahir Dar University, P O Box,79, Bahir Dar, Ethiopia; 2 College of Agriculture and Environmental Sciences, Bahir Dar University, P O Box, 5501 Bahir Dar, Ethiopia

**Keywords:** Adolescent students, Anthropometry, Stunting, Thinness, Ethiopia

## Abstract

**Background:**

Undernutrition among adolescents is a major public health problem in developing nations including Ethiopia. Adolescents need to have good-quantity and good-quality nutrients to cope with this rapid growth and other health risks which increase nutritional demand. This study aimed to assess the prevalence and associated factors of stunting and thinness among school adolescents in Finote Selam Town, Northwest Ethiopia.

**Methods:**

A school-based cross-sectional study among adolescent students aged 10–19 in public schools was carried out in Finote Selam Town from February 5 to March 27, 2018. A total of 397 school adolescents were included in this study. Stratified and simple random sampling techniques were employed to select study subjects. Pretested structured questionnaires were used to collect the data. Data were entered using Epi info version 7 and analyzed using SPSS version 20 and WHO AnthroPlus software. A multivariable logistic regression analysis was employed to identify factors associated with stunting and thinness. Crude and adjusted odds ratios with 95% level significance were used to measure the strength of association, and statistical significance was declared at a *P* value less than 0.05.

**Results:**

The prevalence of stunting and thinness among school adolescents was 21.8% and 16.9%, respectively. About 9.7% of school adolescents experienced both stunting and thinness. Being male (*P* = 0.031), coming from rural area (*P* = 0.046), having a family monthly income of less than $28.37 (*P* = 0.044) and having less than four dietary diversity (*P* = 0.021) were significantly associated with stunting. Early adolescent stage (*P* = 0.034), being male adolescent students (*P* = 0.37), having a family monthly income of less than $28.37 (*P* = 0.016), having a family monthly income between $28.37 and $56.74 (*P* = 0.021) (35.25 Birr = 1 USD) and using well water (*P* = 0.045) were significantly associated with thinness.

**Conclusion:**

Undernutrition was a predominant problem in the study area. Sex, age, place of residence, dietary diversity, sources of drinking water and family monthly income were important factors associated with stunting and thinness among the respondents. Strategies to improve the nutritional status of adolescent students should be given much attention.

## Background

Malnutrition is a major public health problem in both developed and developing nations. Adolescents residing in developing nations, however, suffer more from undernutrition [1]. In many developing countries, under- and over-nutrition occur simultaneously. This phenomenon is referred to as the double burden of malnutrition [2]. Both UN and WHO define adolescence as a segment of the population group age from 10 to 19 years old; it is a transition from childhood to adulthood period that has intense physical, psychosocial and cognitive development. During this period, the final growth spurt occurs; particularly, early adolescence after the first year of life is the critical period of rapid physical growth and changes in body composition, physiology and endocrine. Up to 45% of skeletal growth takes place, and 15–25% of adult height is achieved during adolescence. During the growth spurt of adolescence, up to 37% of total bone mass may be accumulated. Regarding body composition change, girls begin to store fat around the breast, hips and upper arm, but boys start losing fat and develop muscle [3]. Adolescence is a time of changing lifestyles and food habits, changes that affect both nutrient needs and intake [4]. So, it is an opportunity to shape this new behavior adoption [5].

Our world is home to 1.8 billion (24.66%) young people between the ages of 10 and 24, and the youth population is growing fastest in the poorest nations; currently, adolescents make up roughly 20% of the global population [6]. In developing countries, adolescents have higher numbers, about 85% of the demographic age stage, for instance, roughly 26% in Salvador, compared to 14% in the USA [7]. Similarly, in Ethiopia, children and adolescents make up around 48% of the Ethiopian population with adolescents constituting 25% [8].

Pocket studies showed a prevalence of thinness among adolescents, ranging from 19.7% to 83% [9–11], stunting of 16% to 28.5% [11], and overweight or obesity of 2.0% to 16.7% [12, 13] 0.26–30. Gender, age, residence and educational status of the adolescent as well as their parents, age of the child, household wealth index and family size were the most commonly reported socioeconomic determinants in the above studies [12, 14–16].

Most of the worst-performing zones in Ethiopia were found in Amhara region. This could be because the households lack knowledge, attitude and practices (KAP) on how to feed their children and themselves. In this region, it is a common practice to sell more nutritious food items such as legumes (beans, peas and chickpeas), sheep, goat, cattle, milk and milk products since they earn better income by selling these agricultural products rather than feeding these nutritious food items to their children [17, 18]. West Gojjam zone has a high potential in agriculture production like irrigation, particularly fruits and vegetables in addition to livestock and crop production [19, 20]. Finote Selam is the center of the market, and there is a surplus availability of fruits and vegetables. However, there was no study report on nutritional status in the study area. This study will serve as a baseline for further study, important for designing an intervention, and guide policymakers and development planners. This study was mainly focused on assessing the prevalence and various determinants of stunting and thinness among school adolescents in Finote Selam Town, Northwest Ethiopia.

## Methods

### Study design and area

The study design was a school-based, cross-sectional study among adolescent students aged 10–19 in public primary and secondary schools. The study was carried out from February 5 to March 27, 2018, in Finote Selam Town, Ethiopia. There were six primary schools, six junior schools, one high school and one preparatory school in Finote Selam Town. The total number of students from grades 5–12 was 12,289 [21].

### The source and Study Population

All adolescent students (10–19) in Finote Selam Town schools were the source population. Randomly selected adolescent students (10–19) in Finote Selam Town schools were the study population.

### Sample size and sampling procedure

The sample size was calculated using single population proportion formula with the assumptions of the prevalence of thinness among school-going adolescents of Mekelle City, Northern Ethiopia, being 37.8% [22] at a 95% confidence level and 5% margin of error adding 10% as a contingency for non-response rate.

$$n = \left( \frac{z}{d} \right)^{2} xP\left( {1 - p} \right)$$

where *n* = sample size, *Z* = *Z*-score at 95%, CI = 1.96, *p* = 37.8%, *d* = marginal error = 0.05, *n* = (1.96)^2^ * 0.378(1–0.378) = 3.8416 * 0.378*0.622 = 361, (0.05)^2^(0.05)^2^.

By adding 10% non-response rate, the minimum sample size required to estimate the prevalence of stunting and thinness and its associated factors among adolescent students was 361 + 10% (361 + 36) = 397.

To obtain the sample, a stratified random sampling technique was used. The schools were stratified into primary schools, junior schools, high school and preparatory school. Three primary schools, namely Bata, Bakel and Efrata, and three junior schools, namely Edgetber, Bata and Bakel, were selected using simple random sampling from six primary schools and six junior schools, respectively, whereas a high school, namely Finote Selam Secondary School, and a preparatory school, namely Damot Preparatory School, were selected purposively since one high school and one preparatory school were present in Finote Selam Town. The total sample size was distributed proportionally to the schools. The sampling frame was the students’ identification number in their respective schools. The number of students to be included in the study was determined by a simple random sampling method.

### Data collection

To generate the data set used in this study, pretested structured questionnaires were used to collect data by trained data collectors. Explanatory variables were selected after conducting a detailed literature review from the related articles to collect data on the socio-demographic, nutritional and health-related variables [22–24]. Each student was interviewed to obtain information on socio-demographic, nutritional and health-related characteristics of the adolescents’ family.

Anthropometric data were collected by trained data collectors who were health extension workers, and the overall activity was coordinated by the investigator. The age of the adolescents was derived from the school register. Height and weight were measured using a stadiometer and Seca digital scale (Seca Germany), respectively. The weight was recorded to the nearest 0.1 kg. It was calibrated against known weight regularly. Before the real anthropometric data collection, a standardization exercise was performed during the training to capture the technical error of measurement. During the procedure, the subjects wore light clothes and took off their shoes. Height was measured in cm using a portable stadiometer. The height was recorded to the nearest 0.1 cm. The same measure was employed for a given anthropometric measurement to avoid variability.

### Data quality control and management

To ensure the reliability and validity of the study, training was given for the data collectors, the data collection was done by two health extension workers, and closed follow-up was done by the investigator during data collection. The data collectors and investigators participated in the pretesting and standardization of the questionnaires. Problems highlighted during the preliminary study were corrected before the start of the actual survey. Each question was properly coded; continuous supervision was done during the pretest and data collection period by the investigator. Completeness and consistency of recording on the questionnaire sheets were evaluated by the investigator at the end of each working day so that correction measures were taken for the next time.

### Data analyses

Socio-demographic, anthropometric, nutrition and health-related data were entered into the EPI Info version 7 and checked for completeness and consistency, followed by data cleaning and editing on EPI Info. Then, the data were analyzed by using SPSS (Statistical Package for Social Sciences), Version 20 software. WHO AnthroPlus software was used for assessing the growth of adolescents [25]. Descriptive statistics using frequencies and proportions were used to present the study results. For anthropometric data analysis, if the height-for-age Z-score is below minus two standard deviations (− 2 SD) from the median of the reference population, then the child is considered as stunted, and if the BMI-for-age Z-score is below minus two standard deviations (− 2 SD) from the median of the reference population, then the child is thin. Dietary Diversity Score was measured by counting the food items consumed within the previous 24 h and was categorized as poor (consumed < 4 food groups) and good dietary diversity scores (consumed ≥ 4 food groups) [26].

The odds ratio with a 95% confidence interval was used for checking the strength of associations between the outcome variables and independent variables. Bivariate logistic regression was performed, and a variable with a *P* value of less than 0.25 was transported into a multivariable binary logistic regression analysis to identify the determinants of undernutrition of school adolescents. Finally, variables with *P* values < 0.05 in the multivariable logistic regression model were taken as statistically significant [27].

## Results

### Socio-demographic characteristics of study participants and their Family

From a total of 397 adolescent students who were selected as a sample, with a 100% response rate, 397 study participants were involved in this study. Among these, 47 (11.8%) were early adolescents, 151 (38.0%) were mid-adolescents and 199 (50.1%) were late adolescents. Males constituted 249 (62.7%), whereas females constituted 148 (37.3%). Of the participants, 108 (27.2%) were from primary schools, 132 (32.2%) were from junior schools, 116 (29.2%) were from high school and others 41(10.3%) were attending preparatory school. Of the total participants, 195 (49.1%) were from urban areas, whereas 202 (50.9%) were from rural areas. Regarding the family size, 173 (43.6%) and 224 (56.4%) were < 5 family members and ≥ 5 family members, respectively. From the total participants, 257 (64.7%) had functional latrine, whereas 140 (35.3%) did not have a functional latrine.

### Anthropometric results

The minimum and maximum heights of study subjects were 127.50 cm and 186.70 cm, respectively. The mean ± SD overall height of the participants was 158 ± 10.67 cm. Similarly, the minimum and maximum weights of study participants were 20.5 kg and 68 kg, respectively. The mean ± SD overall weight of the participants was 45.99 ± 10.09 kg. The mean heights of boys and girls were 158.99 ± 12.24 cm and 156.31 ± 6.94 cm, respectively. Similarly, the mean weights of boy and girl adolescents were 45.1 ± 10.97 kg and 47.53 ± 8.17 kg, respectively. The mean age of the study participants was 15.54 years (15.54 ± 2.41 SD).

### Prevalence of thinness

The overall prevalence of stunting and thinness among school adolescents at Finote Selam Town was 21.8% and 16.9%, respectively. About 9.7% of school adolescents presented with both stunting and thinness.

**Fig. 1:**
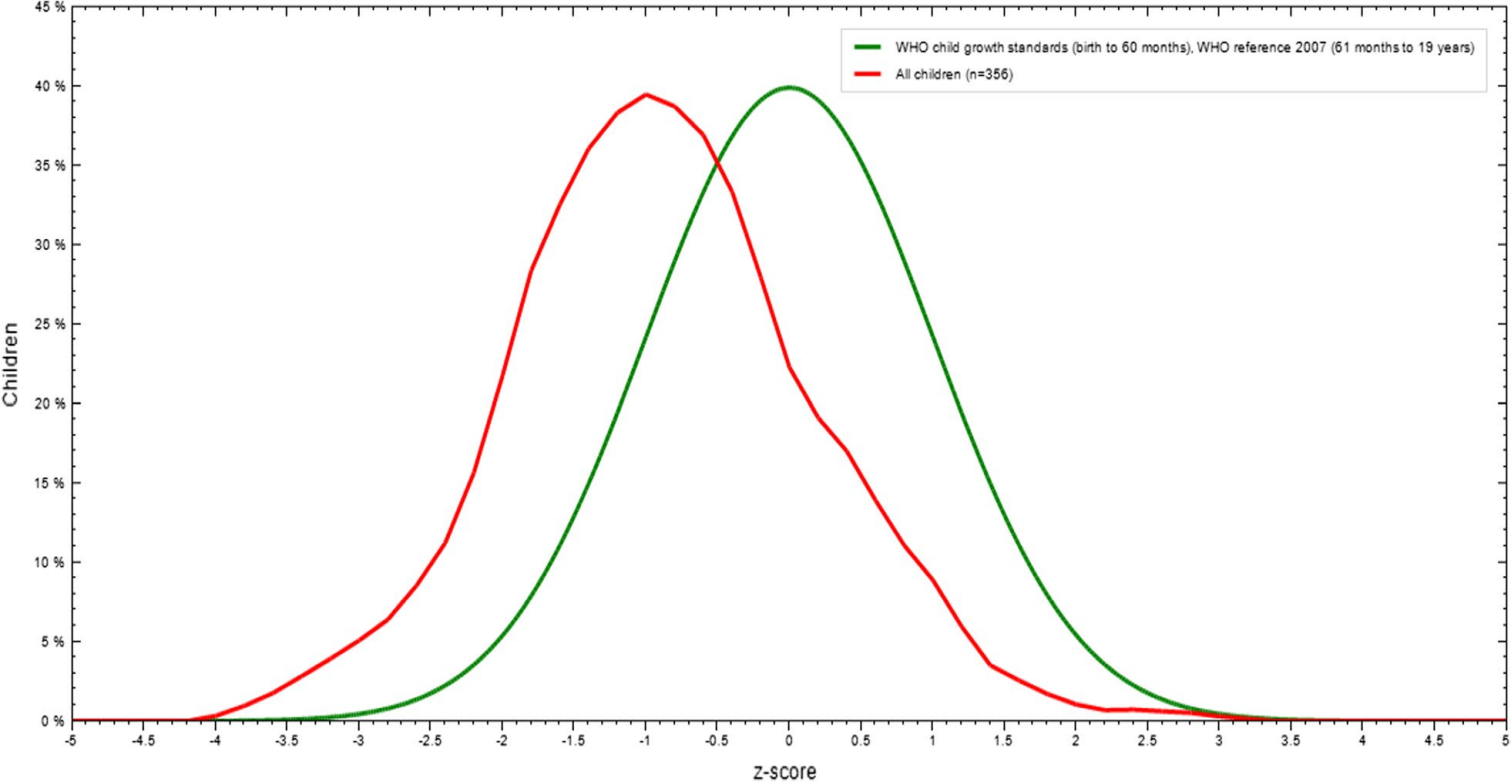
Height-for-age Z-score of adolescent students in Finote Selam Town, Northwest Ethiopia

The mean Z-score of height-for-age was − 1.02 which revealed the distribution of HAZ (Fig. 1).

**Fig. 2:**
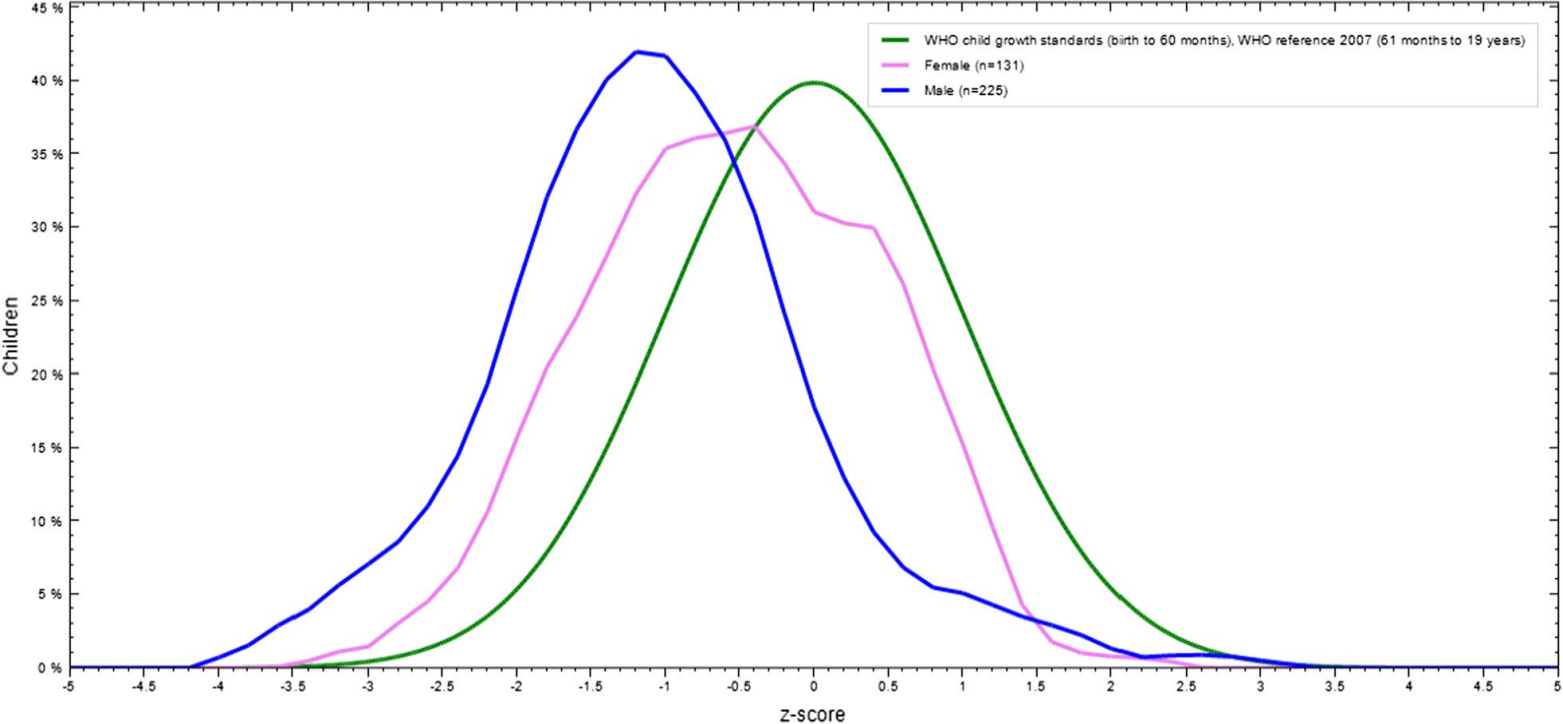
Height-for-age Z-score of adolescent students by sex in Finote Selam Town, Northwest Ethiopia

The mean Z-scores of height-for-age among boys and girls were -1.07 and -0.64 that showed the distribution of HAZ, respectively (Fig. 2).

**Fig. 3:**
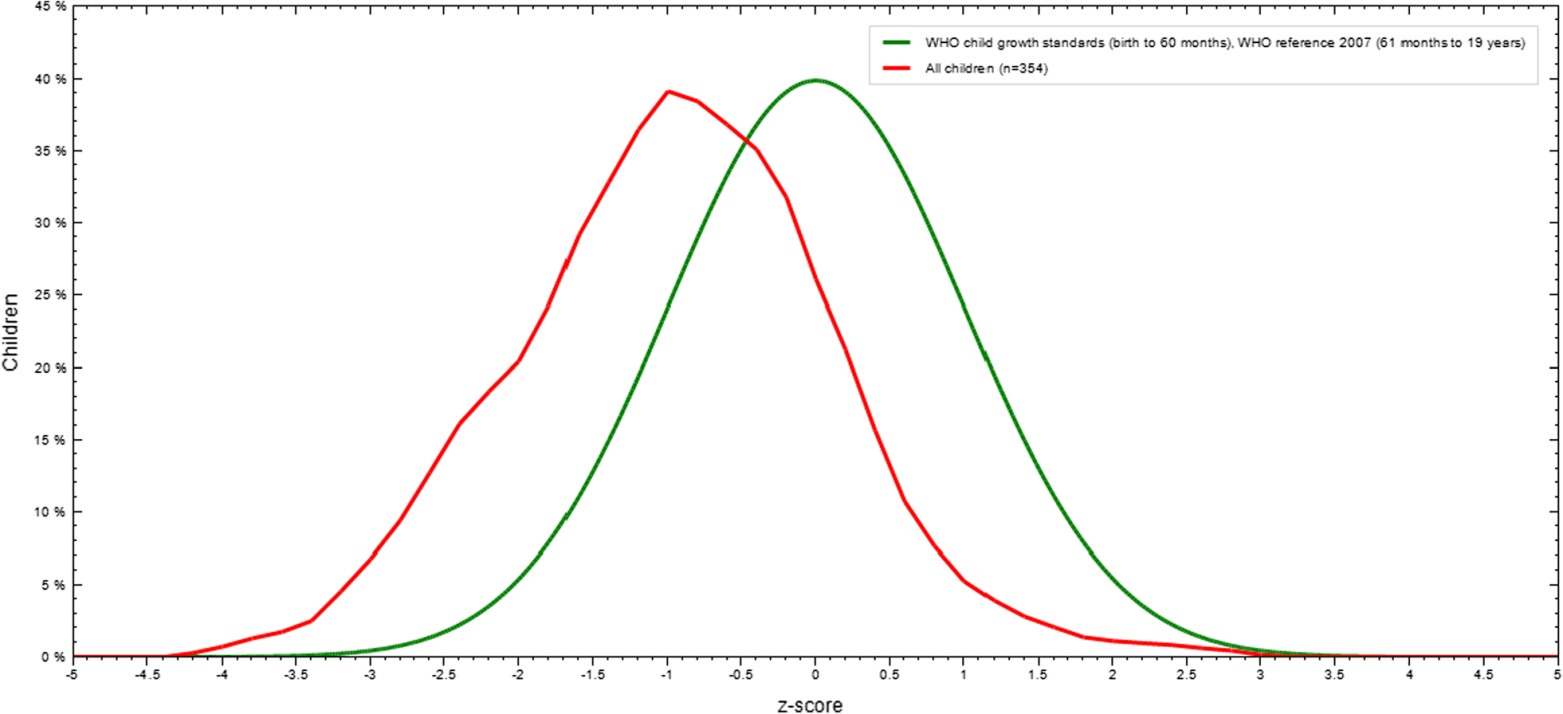
BMI-for-age Z-score of adolescent students in Finote Selam Town, Northwest Ethiopia

The mean Z-score of BMI-for-age of all adolescents was -1.13 which revealed the distribution of BAZ (Fig. 3).

**Fig. 4:**
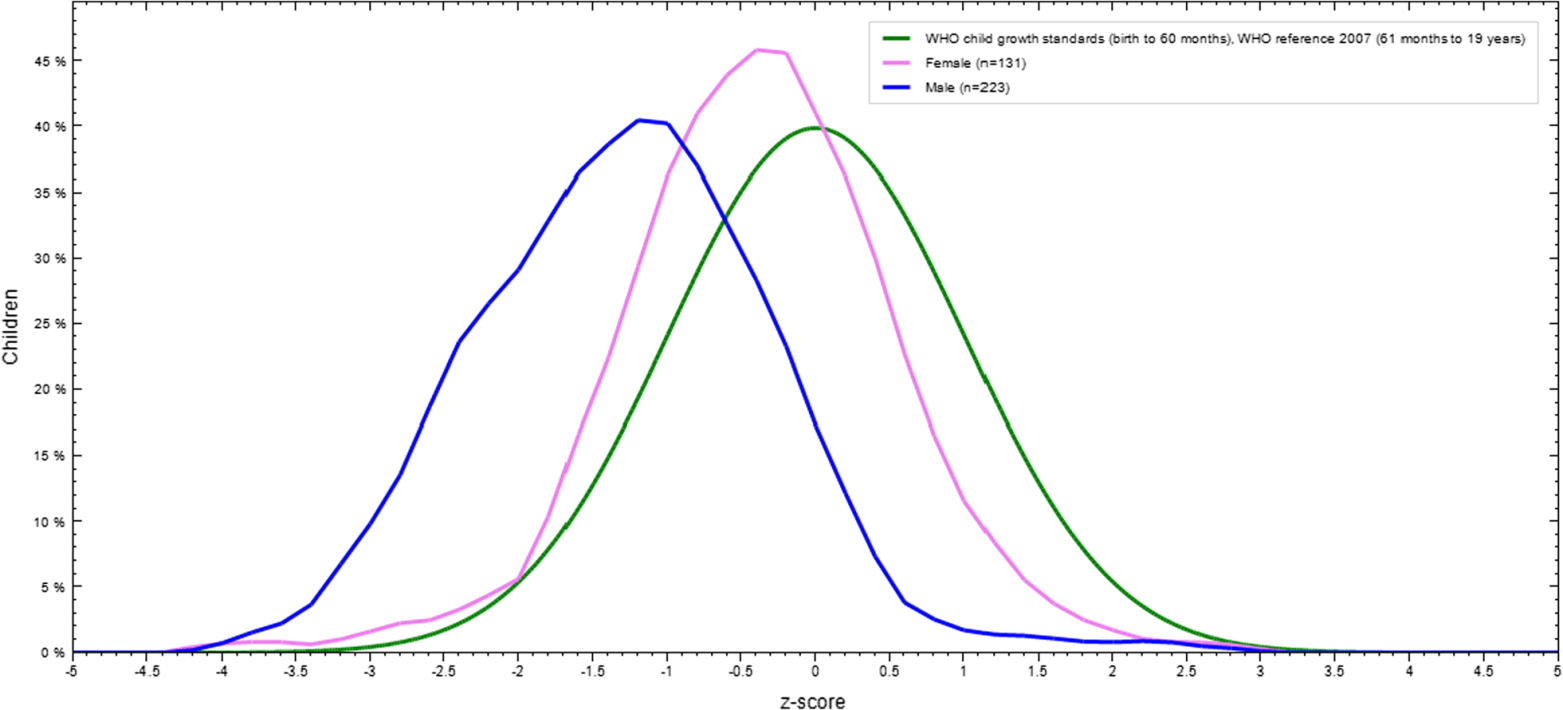
BMI-for-age Z-score of by sex of adolescent students in Finote Selam Town, Northwest Ethiopia

The mean Z-scores of BMI-for-age among boys and girls were -1.29 and -0.51 that showed the distribution of BAZ, respectively (Fig. 4).

## Factors associated with stunting and thinness

### Associated factors of stunting

Identifying determinants associated with stunting is important to take nutritional intervention in the study area. The results revealed that sex, place of residence, family monthly income and dietary diversity were significantly associated with stunting. The results of the adjusted analysis showed that students in the early and middle adolescence were 2.34 [AOR = 2.34; 95% CI 0.81, 6.78] and 2.01 [AOR = 2.01; 95% CI 0.87, 4.61] times more likely to be stunted as compared to late adolescent students, respectively. Male students were 2.27 times more likely to be stunted compared to female students [AOR = 2.66; 95% CI 1.32, 8.13]. The students who came from rural areas were 2.38 times more likely to be stunted compared to urban students [AOR = 2.38; 95% CI 109, 5.18]. The students from families receiving a monthly income of less than $28.37 or $28.37 -$56.74 were 2.61 [AOR = 2.61; 95% CI 1.04, 6.50] and 1.23 [AOR = 1.23; 95% CI 0.45, 3.38] times more likely to be stunted compared to families making over $56.74 per month, respectively. The students who had less than four dietary diversity were 2.61 [AOR = 2.61; 95% CI 1.04, 6.50] times more likely to be stunted compared to the students who had greater than four dietary diversity (Table 1).

**Table 1:** Bivariate and multivariable logistic regression of factors associated with stunting among adolescent school students, Finote Selam Town, Amhara region, Ethiopia, 2018

Variables	Stunting		COR, 95% CI	AOR, 95% CI	*P* value
	Yes	No			
**Age--group**					
Early adolescent (10–13)	12 (20.7%)	46 (79.3%)	2.75 (1.12, 6.77)	2.34 (0.81, 6.78)	
Mid-adolescent (14–16)	20 (11.8%)	150 (88.2%)	1.75 (0.85, 3.62)	2.01(0.87, 4.61)	
Late adolescent (17–19)	13 (7.8%)	156 (86.7%)	1	1	
**Sex**					
Female	31 (20.5%)	120 (79.5%)	1	1	
Male	66 (26.8%)	180(73.2%)	2.67 (1.41, 7.58)	2.27 (1.32, 8.13)*	0.031
**Grade**					
Grade 5–6	17 (15.5%)	93 (84.5%)	1.96 (0.62, 6.14)	1.17 (0.39, 3.54)	
Grade 7–8	23 (18.7%)	100 (81.3%)	1.63 (0.57, 4.68)	1.56 (0.44, 5.51)	
Grade 9–10	12 (11.4%)	93 (88.6%)	0.70 (0.21, 2.36)	0.68 (0.21, 2.28)	
Grade 11–12	11 (18.6%)	48 (81.4%)	1	1	
**Religion**					
Muslim	15 (37.5%)	25 (62.5%)	1	1	
Orthodox	62 (17.4%)	295 (82.6%)	2.10 (0.66, 6.65)	2.54 (0.72, 8.94)	
**Place of residence**					
Urban	36 (18.3%)	161 (81.7%)	1	1	
Rural	56 (28.0%)	144 (72.0%)	2.41 (1.21, 4.80)	2.38 (1.09, 5.18)*	0.046
**Father's education**					
Illiterate	16 (18.0%)	73 (82.0%)	3.19 (0.30, 3.66)	1.84 (0.61, 5.55)	
Read and write	16 (16.0%)	84 (84.0%)	1.59 (0.53, 4.77)	1.48 (0.16, 13.79)	
Primary school (1–8)	10 (20.0%)	40 (80.0%)	1.47 (0.40, 5.46)	1.31 (0.20, 8.41)	
Secondary school (9–12)	12 (13.0%)	80 (87.0%)	1.35 (0.21, 8.83)	1.12 (0.35, 3.60)	
College and above	9 (13.6%)	57 (86.4%)	1	1	
**Mother's education**					
Illiterate	16 (16.8%)	79 (83.2%)	1.95 (0.22, 17.05)	1.50 (0.50, 4.54)	
Read and write	17 (16.0%)	89 (84.0%)	1.88 (0.23, 15.56)	1.44 (0.48, 4.28)	
Primary school (1–8)	9 (19.1%)	38 (80.9%)	1.84 (0.18, 18.91)	1.11 (0.28, 4.43)	
Secondary school (9–12)	12 (13.5%)	77 (86.5%)	1.79 (0.19, 17.07)	1.03 (0.32, 3.34)	
College and above	9 (15.0%)	51 (85.0)	1	1	
**Father's occupation**					
Daily laborer	13 (19.7%)	53 (80.3%)	1.35 (0.43, 4.27)	1.23 (0.46, 3.22)	
Merchant	19 (15.7%)	102 (84.3%)	1.21 (0.53, 2.76)	1.13 (0.31, 4.05)	
Farmer	14 (16.1%)	73 (83.9%)	1.11 (0.45, 2.79)	1.10 (0.36, 3.45)	
Governmental/NGO employee	17 (13.8%)	106 (86.2%)	1	1	
**Mother’s occupation**					
Daily laborer	6 (46.2%)	7 (53.8%)	6.57 (0.93, 46.23)	8.16 (0.85, 78.30)	
House wife	19 (23.0%)	68 (77.0%)	2.16 (0.82, 5.68)	2.48 (0.85, 7.27)	
Farmer	17 (15.9%)	90 (84.1%)	1.49 (0.56, 3.94)	1.84 (0.54, 6.30)	
Merchant	10 (9.4%)	96 (90.6%)	0.64 (0.21, 1.99)	0.67 (0.19, 2.30)	
Governmental/NGO employee	11 (13.1%)	73 (86.9%)	1	1	
**Family size**					
< 5	30 (16.4%)	153 (83.6%)	1	1	
≥ 5	56 (26.2%)	158 (73.8%)	1.38 (0.65, 2.92)	1.01 (0.53, 1.93)	
**Family monthly income**					
< $28.37	29 (32.6%)	60 (67.4%)	4.07(1.92, 8.64)	2.61 (1.04, 6.50)*	0.044
$28.37 -$56.74	20 (18.3%)	89 (81.7%)	1.28 (0.53, 3.07)	1.23 (0.45, 3.38)	
> $56.74	25 (12.6%)	174 (87.4%)	1	1	
**Dietary diversity**					
< 4	44 (15.5%)	239 (84.5%)	4.07(1.92, 8.64)	2.61 (1.04, 6.50)*	0.021
≥ 4	19 (16.7%)	95 (83.3%)	1	1	
**Source of drinking water**					
Spring water	10 (30.3%)	23 (69.7%)	2.25 (0.59, 8.55)	2.63 (0.59, 11.65)	
Well water	27 (15.1%)	152 (84.9%)	1.52 (0.62, 3.72)	2.12 (0.67, 6.67)	
Public tap water	14 (14.4%)	83 (85.6%)	1.17 (0.41, 3.31)	1.21 (0.38, 3.83)	
Tap water	12 (13.6%)	76 (86.4%)	1	1	
**Presence of functional latrine**					
Yes	36 (14.5%)	213 (85.5%)	1	1	
No	27 (18.2%)	121 (81.8%)	1.13 (0.58, 2.19)	1.08 (0.55, 2.15)	
**Frequency of meals eaten per day**					
Two times	29 (23.4%)	95 (76.6%)	2.02 (1.04, 3.91)	1.89 (0.94, 3.81)	
Three times and above	34 (12.5%)	239 (87.5%)	1	1	
**Illness reported in the last one month**					
Yes	26 (19.7%)	106 (80.3%)	1.26 (0.64, 2.48)	1.18 (0.59, 2.39)	
No	37 (14.0%)	228 (86.0%)	1	1	
**Availability of home garden**					
Yes	38 (18.5%)	167 (81.5%)	1	1	
No	39 (20.3%)	153 (79.7%)	1.41 (1.00, 1.97)	1.06 (0.70, 1.61)	

AOR: adjusted odds ratio; COR: crude odds ratio; CI: confidence interval; * significant *P* value < 0.05; 1 = reference

### Associated factors of thinness

Exploring determinants associated with thinness is important to take nutritional intervention action in the study area. Results of the multivariable binary logistic regression model showed that age, sex, family monthly income and sources of drinking water were significantly associated with thinness. The odds of thinness were 4.81 times higher among adolescent students in the early adolescent stage as compared to adolescent students in the late adolescent stage [AOR = 4.81; 95% CI 1.23, 18.51]. The reason is likely because the younger kids 10 years old, especially boys, have not hit their growth spurt yet. Male adolescent students had 2.13 times higher odds of thinness [AOR = 2.13; 95% CI 1.60, 3.40] compared to female adolescent students. The students who came from rural areas were 2.16 [AOR = 2.61; 95% CI 0.96, 4.87] times more likely to be thin compared to those who came from urban areas. The students from families receiving a monthly income of less than $28.37 or $28.37–$56.74 were 6.54 [AOR = 6.54; 95% CI 3.82, 14.89] and 3.47 [AOR = 3.47; 95% CI 1.15, 7.45] times more likely to be thin compared to families making over $56.74 per month, respectively. Students from households that used well water as their main source of water were 3.82 times more likely to be at risk of being thin than students from households that used tap water supply for human consumption [AOR = 3.82; 95% CI 1.46, 10.04] (Table 2).

**Table 2:** Bivariate and multivariable logistic regression of factors associated with thinness among adolescent school students, Finote Selam Town, Ethiopia, 2018

Variables	Thinness		COR (95% CI)	AOR (95% CI)	*P* value
	Yes	No			
**Age-group**					
Early adolescent (10–13)	17 (36.2%)	30 (63.8%)	5.14 (2.39, 11.08)*	4.81 (1.23, 18.51)*	0.034
Mid-adolescent (14–16)	24 (15.9%)	127 (84.1%)	1.90 (0.99, 3.64)	1.68 (0.57, 4.93)	
Late adolescent (17–19)	18 (9.0%)	181 (91.0%)	1	1	
**Sex**					
Female	4 (2.7%)	144 (97.3%)	1	1	
Male	55 (22.1%)	194 (77.9%)	2.68 (1.88, 3.84)	2.13 (1.60, 3.40)*	0.037
**Grade**					
Grade 5–6	23 (21.3%)	85 (78.7%)	2.57 (0.91, 7.24)	1.77 (0.40, 7.87)	
Grade 7–8	22 (16.7%)	110 (83.3%)	2.08 (0.74, 5.86)	1.33 (0.22, 8.19)	
Grade 9–10	10 (8.6%)	106 (91.4%)	1.01 (0.32, 3.15)	1.06 (0.20, 5.58)	
Grade 11–12	4 (9.8%)	37 (90.2%)	1	1	
**Religion**					
Muslim	1 (4.5%)	21 (95.5%)	1		
Orthodox	58 (15.5%)	317 (84.5%)	3.82 (0.50, 29.18)	3.90 (0.19, 81.94)	
**Place of residence**					
Urban	18 (9.2%)	177 (90.8%)	1	1	
Rural	41 (20.3%)	161 (79.7%)	2.44 (1.35, 4.40)*	2.16 (0.96, 4.87)	
**Father’s education**					
Illiterate	15 (15.8%)	80 (84.2%)	2.79 (0.92, 8.45)	4.06 (0.23, 71.76)	
Read and write	17 (16.8%)	84 (83.2%)	1.97 (0.71, 5.43)	2.73 (0.31, 24, 29)	
Primary school (1–8)	10 (24.4%)	31 (75.6%)	1.96 (0.73, 5.32)	2.36 (0.26, 21.41)	
Secondary school (9–12)	11 (12.0%)	81 (88.0%)	1.44 (0.51, 4.10)	0.80 (0.5, 12.90)	
College and above	6 (8.8%)	62 (91.2%)	1	1	
**Mother’s education**					
Illiterate	17 (16.7%)	85 (83.3%)	2.38 (0.77, 7.39)	1.78 (0.86, 3.65)	
Read and write	16 (14.7%)	93 (85.3%)	1.86 (0.68, 5.07)	1.40 (0.83, 2.34)	
Primary school (1–8)	9 (26.5%)	25 (73.5%)	1.50 (0.55, 4.09)	1.29 (0.61, 2.71)	
Secondary school (9–12)	11 (12.4%)	78 (87.6%)	1.33(0.47, 3.81)	1.15 (0.66, 1.97)	
College and above	6 (9.5%)	57 (90.5%)	1	1	
**Father’s occupation**					
Daily laborer	8 (14.0%)	49 (86.0%)	1.20 (0.47, 3.07)	1.1 (0.55, 21)	
Farmer	26 (21.8%)	93 (78.2%)	2.27 (1.12, 4.61)*	1.55 (0.45, 5.29)	
Merchant	11 (12.9%)	74 (87.1%)	1.18 (0.50, 2.75%)	1.52 (0.55, 4.26)	
Governmental/NGO employee	14 (10.3%)	122 (89.7%)	1	1	
**Mother’s occupation**					
Daily laborer	1 (20.0%)	4 (80.0%)	1.86 (0.19, 18.55)	2.10 (0.11, 38.74)	
Farmer	21 (24.4%)	65 (75.6%)	2.74 (1.16, 6.46)*	2.16 (0.62, 7.51)	
House wife	15 (12.6%)	104 (87.4%)	1.33 (0.55, 3.23)	2.46 (0.58, 10.47)	
Merchant	13 (12.1%)	94 (87.9%)	1.24 (0.51, 3.04)	1.52 (0.43, 5.36)	
Governmental/NGO employee	9 (11.2%)	71 (88.8%)	1	1	
**Family size**					
< 5	17 (9.8%)	156 (90.2%)	1	1	
≥ 5	42 (18.8%)	182 (81.2%)	2.43 (1.32, 4.45)*	1.90 (0.80, 4.51)	
**Dietary diversity**					
< 4	44 (15.5.9%)	240 (84.5%)	1.36 (0.71, 2.61)	1.15 (0.67, 1.97)	
≥ 4	18 (15.9%)	95 (84.1%)	1	1	
**Family monthly income**					
< $28.37	29 (37.7%)	48 (62.3%)	6.10 (3.09, 12.04)*	11.54 (3.82, 34.89)*	0.016
$28.37 -$56.74	14 (13.1%)	93 (86.9%)	1.71 (0.80, 3.64)	3.47 (1.15, 10.45)*	0.021
> $56.74	16 (7.5%)	197 (92.5%)	1	1	
**Source of drinking water**					
Well water	39 (21.3%)	144 (78.7%)	4.53 (1.71, 11.98)	3.82 (1.46, 10.04)*	0.045
Spring water	13 (12.3%)	93 (87.7%)	3.37 (0.88, 12.89)	2.56 (0.87, 7.56)	
Public tap water	2 (7.7%)	24 (92.3%)	1.88 (0.23, 15.49)	1.46 (0.26, 8.09)	
Tap water	5 (6.1%)	77 (93.9%)	1	1	
**Presence of functional latrine**					
Yes	38 (14.8%)	219 (85.2%)	1	1	
No	21 (15.0%)	119 (85.5%)	1.08 (0.49, 2.38)	1.05 (0.59, 1.87)	
**Frequency of meals eaten per day**					
Two times	19 (16.0%)	100 (84.0%)	1.17 (0.64, 2.13)	1.01 (0.53, 1.91)	
Three times and above	40 (14.4%)	238 (85.6%)	1	1	
**Illness reported in the last one month**					
Yes	19 (14.5%)	112 (85.5%)	1.06 (0.57, 1.96)	1.03 (0.57, 1.87)	
No	40 (15.0%)	226 (85.0%)	1	1	
**Availability of home garden**					
Yes	50 (17.4%)	238 (82.6%)	1	1	
No	9 (8.3%)	100 (91.7%)	1.60 (1.01, 2.54)	1.15 (0.66, 1.99)	

AOR: adjusted odds ratio; COR: crude odds ratio; CI: confidence interval; * significant *P* value < 0.05; 1 = reference

## Discussion

In this study, the nutritional status and associated factors among school adolescents in Finote Selam Town, Northwest Ethiopia, were assessed. This study revealed that the prevalence of stunting among adolescent students at Finote Selam Town was 21.8% which was higher than the results documented in Jimma Zone, Southwest Ethiopia (16%) [27], in Tehuledere District, Northeast Ethiopia (15.5%) [28], and Addis Ababa (7.2%) [13], but lower than the finding reported in Wukro, Northern Ethiopia (28.5%) [23]. This could be because the households lack knowledge, attitude and practices (KAP) on how to feed their children and themselves [29]. Several studies in other African countries including Burkina Faso (12%) [30] and Mongolia (15.6%) [31] had reported a lower prevalence of stunting. Although the feeding practices for children in Ethiopia are generally poor, the complementary feeding practices are worse in Amhara region than in other parts of Ethiopia as a whole [32]..

The prevalence of thinness was 14.9% and lower than the studies in Mekelle City (37.8%) [9], Ambo City (27.5%) [33], Wukro District (26.1%) [23] and Jimma Zone (80.8%) [34], however, higher than the study conducted in Addis Ababa (6.2%) [9]. This divergence might be due to differences in socioeconomic background, dietary habits and type of meals among the cities. Findings in other African countries including Burkina Faso (13.7% [35], Asembo and Mumias, Kenya (15.6%) [36], Tunisia (13.0%) [37], and Tamale Metropolis, Ghana (10.0%) [38]. This divergence might be because in this region, it is a common practice to sell more nutritious food items such as legumes (beans, peas and chickpeas), sheep, goat, cattle, milk and milk products since they earn better income by selling these agricultural products rather than feeding these nutritious food items to their children [39].

Among the factors considered in this study, sex, place of residence, dietary diversity and family monthly income were associated with stunting. Male students were 2.27 times more likely to be stunted compared to female students, because male puberty hit later than female. Female puberty usually starts between ages 9 and 11. In males, puberty usually starts around age 11 [40]. This finding was supported by the result of similar studies conducted in Wukro, Northern Ethiopia [23] Chiro Town, West Hararge [41] and Tehuledere District, Northeast Ethiopia [28]. The increased risk for stunting seen among males in this study suggests that males may experience greater exposure to risks for chronic undernutrition. It appears possible that these earlier gender differences in stunting are persisting into adolescence. This compensatory growth may be occurring later in males since they undergo puberty later than females [42]. The students who came from rural areas were 2.38 times more likely to be stunted compared to urban students. This finding was similar to the finding in Wukro [23] and Adwa town, Northern Ethiopia [43]. However, ample evidence documents that urban children generally have better nutritional status than their rural children. The environment, choices and opportunities of urbanites differ greatly from those of rural dwellers' from employment conditions to social and family networks to access healthcare and other services [44]. This association might also be related to differences in dietary practices, where rural diets are predominantly cereal-based, as well as lower literacy rates and limited access to water, sanitation and hygiene facilities in rural areas [45]. The students from families receiving a monthly income of less than $28.37 or $28.37–$56.74 were 2.61and 1.23 times more likely to be stunted compared to families making over $56.74 per month, respectively. This is due to underlying reason of less affordability of healthcare, quality nutrition and hygiene rather than rich families [46]. The possible reason is also due to the positive correlation between income level and nutrition status of adolescents; higher income could mean a stronger purchasing power for better-quality foods, while limited income restricts access to nutrient-dense foods [47]. The odds of stunting was 2.61 times higher among adolescent students who had a score of < 4 dietary diversity compared to adolescent students who reported greater than or equal to four dietary diversity. This finding is supported by the study conducted in Adama City, Ethiopia [48]. This is because when the intake of dietary diversity is high, adolescents receive adequate energy and important nutrients that play an integral role in growth and development [49].

According to the multivariable logistic regression analysis, sex, age, sources of drinking water and family monthly income were significantly associated with thinness. The odds of thinness was 4.81 times higher among adolescent students in the early adolescent age stage as compared to adolescent students in the late adolescent age stage. This finding was in agreement with findings from Adwa Town, Northern Ethiopia [50], and community-based nutrition implementing districts, Amhara region, Ethiopia [51]. The possible reason is increased growth spurt in the early adolescent stage as compared to the late adolescent stage [43]. Also, the scarcity of information on nutritional status during middle childhood and early adolescence is particularly remarkable [52]. On the other hand, as age increases, adolescents become more matured. Male adolescent students had 2.13 times higher odds of thinness compared to female adolescent students. This study was in line with the studies conducted in Mekelle City, Northern Ethiopia [9], Jimma Zone, Southwest Ethiopia [34] and Wukro, Northern Ethiopia [23], which confirmed that males were more affected due to thinness than girls. This might be due to variation of maturation time in boys and girls, for which girls reached maturation earlier than boys. The students from families surviving on less than $28.37, or $28.37–$56.74 monthly were 6.54 and 3.47 times more likely to be experiencing thinness compared to those with family monthly income greater than $56.74. This is likely due to the positive correlation between income level and the nutrition status of adolescents. As income rises, families are more likely able to afford more than two times of meals per day. Finally, students from households that used well water as a main source of water were 3.82 times more likely to be at risk of being thin than students from households that used tap water for human consumption. This finding was in line with findings from Adwa Town, Northern Ethiopia [43]. This might be because impure water is a vehicle for intestinal parasites, which leads to loss of appetite, and this leads to poor nutritional status. The repeated infection causes depressed immunity, meaning that the outcome and duration of the disease is more severe and contributing to the poor nutritional status of the adolescents.

## Conclusions

The study showed that undernutrition is a prevalent problem in the study area. Consistent with this result, the mean Z-scores of height-for-age were higher in boys than girls. Sex, age, place of residence, dietary diversity, sources of drinking water and family monthly income were found to be important factors associated with stunting and thinness among the respondents. Strategies to improve the nutritional status of adolescent students should be given much attention.

### Limitations of the study

A limitation was the use of a cross-sectional study design which could only generate a hypothesis regarding the role of independent variables on the nutritional status of adolescent students but not their cause-and-effect relationships; the age of the students obtained from school records might be underestimated; there may also be measurement bias during anthropometric measurements. The research also failed to explore other parental socio-demographic and socioeconomic characteristics. BMI alone cannot show whether a person’s weight is healthful, but using it in combination with other indicators can provide a more complete picture.

## Abbreviations

AOR: Adjusted odds ratio
BAZ: BMI-for-age Z-score
BMI: Body Mass Index
CM: Centimeter
CV: Confidence interval
COR: Crude odds ratio
CSA: Central Statistics Agency
CV: Confidence interval
EDHS: Ethiopian Demography and Health Survey
HAZ: Height-for-age Z-score
HFA: Height-for-age
SD: Standard deviation
SPSS: Statistical Package for Social Sciences
UN: United Nations
WHO: World Health Organization

## Declarations

### Availability of data and materials

Data will be available upon request from the corresponding author via email dakebede10@gmail.com.
